# Microstructural brain abnormalities, fatigue, and cognitive dysfunction after mild COVID-19

**DOI:** 10.1038/s41598-024-52005-7

**Published:** 2024-01-19

**Authors:** Lucas Scardua-Silva, Beatriz Amorim da Costa, Ítalo Karmann Aventurato, Rafael Batista Joao, Brunno Machado de Campos, Mariana Rabelo de Brito, José Flávio Bechelli, Leila Camila Santos Silva, Alan Ferreira dos Santos, Marina Koutsodontis Machado Alvim, Guilherme Vieira Nunes Ludwig, Cristiane Rocha, Thierry Kaue Alves Silva Souza, Maria Julia Mendes, Takeshi Waku, Vinicius de Oliveira Boldrini, Natália Silva Brunetti, Sophia Nora Baptista, Gabriel da Silva Schmitt, Jhulia Gabriela Duarte de Sousa, Tânia Aparecida Marchiori de Oliveira Cardoso, André Schwambach Vieira, Leonilda Maria Barbosa Santos, Alessandro dos Santos Farias, Mateus Henrique Nogueira, Fernando Cendes, Clarissa Lin Yasuda

**Affiliations:** 1 Brazilian Institute of Neuroscience and Neurotechnology (BRAINN), University of Campinas, Campinas, Brazil; 2 Department of Neurology, Clinics Hospital, University of Campinas, Campinas, Brazil; 3 Institute of Mathematics, Statistics and Scientific Computing, University of Campinas, Campinas, Brazil; 4 Molecular Genetics Laboratory, Faculty of Medical Sciences, University of Campinas, Campinas, Brazil; 5 Autoimmune Research Lab, Institute of Biology, University of Campinas, Campinas, Brazil; 6 Department of Radiology, Clinics Hospital, University of Campinas, Campinas, Brazil

**Keywords:** Neuroscience, Medical research, Neurology, SARS-CoV-2, Image processing

## Abstract

Although some studies have shown neuroimaging and neuropsychological alterations in post-COVID-19 patients, fewer combined neuroimaging and neuropsychology evaluations of individuals who presented a mild acute infection. Here we investigated cognitive dysfunction and brain changes in a group of mildly infected individuals. We conducted a cross-sectional study of 97 consecutive subjects (median age of 41 years) without current or history of psychiatric symptoms (including anxiety and depression) after a mild infection, with a median of 79 days (and mean of 97 days) after diagnosis of COVID-19. We performed semi-structured interviews, neurological examinations, 3T-MRI scans, and neuropsychological assessments. For MRI analyses, we included a group of non-infected 77 controls. The MRI study included white matter (WM) investigation with diffusion tensor images (DTI) and functional connectivity with resting-state functional MRI (RS-fMRI). The patients reported memory loss (36%), fatigue (31%) and headache (29%). The quantitative analyses confirmed symptoms of fatigue (83% of participants), excessive somnolence (35%), impaired phonemic verbal fluency (21%), impaired verbal categorical fluency (13%) and impaired logical memory immediate recall (16%). The WM analyses with DTI revealed higher axial diffusivity values in post-infected patients compared to controls. Compared to controls, there were no significant differences in the functional connectivity of the posterior cingulum cortex. There were no significant correlations between neuropsychological scores and neuroimaging features (including DTI and RS-fMRI). Our results suggest persistent cognitive impairment and subtle white matter abnormalities in individuals mildly infected without anxiety or depression symptoms. The longitudinal analyses will clarify whether these alterations are temporary or permanent.

## Introduction

Studies have consistently reported neurological manifestations of COVID-19^1^. While most individuals will recover from respiratory symptoms, the course of post-COVID-19 fatigue and cognitive dysfunction is uncertain. One French study identified the dysexecutive syndrome in 15/45 (33%) patients with severe COVID-19 infection^2^. Another Chinese study recruited 29 patients (after hospitalisation) and reported cognitive dysfunction after their recovery^3^. A third Swiss study with 121 patients also detected executive and amnestic deficits in patients after moderate and severe COVID-19^4^. Symptoms of anxiety, depression, and alterations of different cognitive domains have also been described, some persisting over 2 years^5–10^. However, there is a limitation in the present understanding of the post-infectious neurological and cognitive dysfunctions (including the nature, duration, and pathophysiology) in individuals who recovered from COVID-19^11^, especially those who had a mild infection and who did not require hospitalisation.

Although the neuroinvasion of SARS-CoV-2 has been demonstrated with confirmation of the virus in some brain autopsies^12,13^, the neural mechanisms underlying both neurological and neuropsychiatric symptoms (acute and chronic) remain unclear. Neuroimaging analyses have been used to investigate structural and functional brain changes after infection by SARS-CoV-2; however, conflicting results have been reported regarding white matter (WM)^14–16^, and grey matter (GM) changes^13,14,17^. In terms of white matter, one analysis of hospitalized patients in China reported higher levels of fractional anisotropy in the patient group, 3 months after SARS-CoV-2 infection^14^, but it did not include neuropsychological tests. On contrary, another study with 22 hospitalized patients revealed reduced fractional anisotropy of the corpus callosum. In a recent analysis of 97 patients (95% non-hospitalized) we did not identify changes of WM diffusivities or cognitive dysfunction^16^. The recent analyses of 58 hospitalized individuals (without formal cognitive evaluation or inclusion of healthy controls) described increased values of axial, radial, and mean diffusivities in the group with cognitive complaints, compared to the group without complaints; this study also revealed alterations of functional connectivity between the two groups^18^. Regarding cerebral functional connectivity (FC), the default mode network (DMN) is one of the most studied brain networks. Alterations of DMN have been associated with different diseases (i.e., Alzheimer’s disease, epilepsy, and others) and symptoms commonly observed in individuals with post-COVID conditions (PCC), such as sleepiness^19^ and fatigue^20^. Although some resting-state fMRI studies showed alterations of FC after COVID-19, fewer focused exclusively on mildly infected subjects, on the DMN, or investigated the relationship between DMN and excessive fatigue and somnolence^21–23^.

Several studies have evaluated symptoms and signs after the initial COVID infection; however, most have not applied official definitions of the PCC (such as those presented by the US Center for Disease Control and Prevention (CDC), and the World Health Organization (WHO)^24^). The WHO defines “post-COVID condition” as only after 3 months from the acute infection^25,26^, in contrast, the Center for Disease Control defines 4 weeks^26^, instead of 3 months^26,27^. Added to the differences about definitions of PCC, most neuroimaging studies have focused on patients with severe acute infection (which may result in brain alterations and cognitive dysfunction independently of COVID infection^28,29^), or included subjects with mild and severe infections with and without symptoms of anxiety and depression^21,23^. Unfortunately, the heterogeneity of studies (i.e. lack of a consistent definition of PCC^26^ and analyses of severe patients) compromises the comparisons of results and conclusions related to the impact of SARS-CoV-2 in the nervous system^24^.

In this study, we used the CDC’s criteria for PCC^26,27^ and analysed a group of non-hospitalized individuals who had confirmed infection. We did not include individuals with current symptoms of anxiety or depression (nor those with a history of anxiety or depression) to avoid the biases related to brain changes^30^ and cognitive dysfunction^31^ associated with symptoms of anxiety and depression. We investigated clinical symptoms, cognitive function, symptoms of fatigue and somnolence. Additionally, we searched for white matter abnormalities and changes in DMN’s functional brain connectivity.

## Methods

### Sample and study design

All our methods were carried out in accordance with relevant guidelines and regulations and all subjects signed an informed consent form to participate. The Research Ethics Committee of the University of Campinas approved this study (Certificate of Ethical Appreciation Presentation—CAAE 31556920.0.0000.5404).

#### Subjects

We conducted a cross-sectional data analysis from a longitudinal observational study designed to evaluate post-acute neurological symptoms and neuroimaging alterations related to COVID-19^13^. We used social media to advertise our study with an online questionnaire^32^ (Supplementary Table 2). We successively recruited the responders who presented a confirmed diagnosis of COVID-19 (independent of the severity of acute COVID-19 status) to visit our centre and to perform the four steps of the complete protocol (on the same day): a personal semi-structured interview (with planned and open questions) and neurological examination (performed by certified neurologists), a 3T MRI acquisition, a brief neuropsychological evaluation and blood sample collection at the University of Campinas Hospital.

The COVID-19 diagnoses were based on polymerase chain reaction (PCR) tests or confirmed IgM or IgG antibodies.

According to the inclusion and exclusion criteria described below, we included 114 patients with (details are presented in Supplementary Fig. 1). This study’s inclusion criteria were: COVID-19 diagnosis (confirmed with PCR test, IgM or IgG antibodies) with or without specific (or unspecific) complaints, MRI acquisition after 4 weeks after COVID-19 diagnosis (as per the CDC definition^27^), minimum age of 18 years old, no hospitalization, normal neurological exam, and normal 3T MRI (at visual inspection).

The exclusion criteria include MRI acquisition within less than 4 weeks since the acute infection, abnormal neurological examination, MRI acquisition with 8 channels head coil, history of neuropsychiatric disorders (including depression or anxiety) or current symptoms of anxiety (defined by a BAI of 11 or higher) or depression (defined by a BDI of 14 or higher) at the time of MRI acquisition.

As a control group, we recruited 77 healthy volunteers (median age 36 years (range 20–68)) from the same environment as the patients (without a history of neurological or psychiatric disorders). These individuals had never presented COVID-19-related symptoms (fever, anosmia, cough, or dysgeusia) and never tested positive for COVID-19. For the twenty subjects scanned in 2020 and 2021 (before the national vaccination campaign), we performed a rapid test immediately before the MRI scan (immunochromatographic essay 2019-nCoV IgG/IgM Combo Test Card—MP Biomedicals Germany GmbH™) to confirm they were not infected.

#### Neuropsychological evaluation

Due to the uncertainties related to cognitive impairment associated with SARS-CoV-2, we performed an exploratory neuropsychological evaluation of recovered individuals. We intentionally selected tests to evaluate different cognitive domains including: *global cognition* (Mini-Mental State Examination [MMSE]^33^), *language* (the Verbal Categorical Fluency Test^34^ and the Phonemic Verbal Fluency Test^35^), *verbal episodic memory* (the Logical Memory subtest from the Wechsler Memory Scale [WMS-R]^36^), *visuoconstruction and visuospatial episodic memory* (the Rey Complex Figure Test^37^), *manual dexterity* (9-Hole Peg Test^38^), *processing speed* (Five Digit Test—Reading and Counting), *selective attention* (FDT—Choice), *inhibitory control* (FDT-Inhibition), *alternate attention* and *cognitive flexibility* (FDT—Alternation and Flexibility)^39^, and *sustained attention, perceptual tracking and motor skills* (the Color Trail Test [CTT]—part A and B^40^). Although we have organized the neuropsychological tests according to the main cognitive functions assessed, it is known that they can involve other cognitive functions. For example, executive functions are assessed with categorical and phonemic verbal fluency tests, as well as with Color Trail Tests^41^. We have included a detailed description in the Supplementary Material (the “Neuropsychological instruments” section).

We calculated the z-scores for the results of the neuropsychological tests based on the Brazilian standard and scaled scores. We controlled for the effects of age or schooling in a separate analysis using multiple linear regression residuals when normative data covered only one of these variables. For each test, the function was categorised as *preserved* (z-score >  − 0.66, including average, high-average, above-average, and exceptionally high scores), *low-average* score (z-score between − 0.7 and − 1.26), *below-average* score (z-score between − 1.32 and − 1.82), and *exceptionally low* score (z-score <  − 1.96)^42^.

We quantified anxiety symptoms with the Beck Anxiety Inventory (BAI), and symptoms of depression with the Beck Depression Inventory II (BDI-II). We also investigated fatigue with the Chalder Fatigue Questionnaire (CFQ-11)^43,44^ and excessive daytime sleepiness with the Epworth Sleepiness Scale (ESS)^45^. Added to these validated questionnaires, patients self-reported subjective complaints after COVID-19 infection during the semi-structured interview. Details of these tests are described in the Supplementary Material, the “Neuropsychological instruments” section.

#### MRI acquisitions

All individuals underwent 3T MRI (Phillips Achieva) with a 32-channels head coil. We acquired volumetric (3D) T1-weighted scans, resting-state functional MRI (fMRI) scans using echo-planar imaging (EPI) sequences, and diffusion tensor imaging (DTI) scans with 32 directions.

*T1-weighted images* were acquired from each subject using isotropic voxels of 1 mm, acquired in the sagittal plane, 1 mm thick, no gap, flip angle = 8°, TR = 7.0 ms, TE = 32 ms, matrix = 240 × 240, FOV = 240 × 240 mm^2^. *Resting-state images* were acquired as echo planar images (EPI) with voxel sizes of 3 × 3 × 3 mm^3^, acquired on the axial plane with 40 slices, no gap, flip angle = 90°, TR = 2 s, TE = 30 ms in a 6-min scan resulting in 180 dynamics and FOV = 240 × 240 mm^2^. *Diffusion weighted* were acquired from each subject through spin-echo single-shot EPIs sequences (TE = 61 ms; TR = 8500 ms, single-shell, max b-factor = 1000 mm/s^2^, flip-angle = 90°, 32 directions) with 70 axially oriented 256 × 256 planes resulting in isotropic 2 × 2 × 2 mm^3^ voxels interpolated to 1 × 1 × 2 mm^3^ voxels.

The specific protocol for MRI acquisitions of COVID-19 individuals is included in the “MRI protocol” section of the Supplementary File.

#### MRI analyses

##### Diffusion data analysis with TBSS

Data were pre-processed using the recommended pipeline from the ENIGMA consortium^46^. Briefly, raw data were processed with different toolboxes by denoising, Gibbs ring artifact removal, movement and eddy current correction, bias field correction and tensor fitting for the extraction of fractional anisotropy (FA), axial diffusivity (AD), mean diffusivity (MD) and radial diffusivity (RD) maps.

FA maps were normalized to standard FMRIB58_FA space, averaged and skeletonized by the TBSS algorithm^47^. Subject’s FA, AD, MD and RD data were projected into the mean FA skeleton for voxel-wise statistical analysis (comparisons between patients and controls) with threshold-free cluster enhancement (TFCE)^48^ using age and sex as covariates. The correction for multiple comparisons was performed using TFCE^48^ as implemented in FSL’s *randomize*^49^ with parameters adjusted for the skeletonized data (H = 2, E = 1, 26-voxel-connectivity) and a null distribution estimated using 10,000 random permutations of the data. Details of the analysis pipeline are presented in the Supplementary Material.

##### Functional connectivity (FC)

Given the uncertainty about the changes in cerebral functional FC after recovery from COVID-19 infection, we focussed the analyses on a well-known network, the default mode network (DMN)^50^. We investigated changes in the typical pattern observed in healthy volunteers, as well as the relationship between the DMN and fatigue^51^ and daytime sleepiness.

We performed the pre-processing steps and FC analysis with the UF^2^C toolbox (https://www.lniunicamp.com/uf2c) within SPM12 (http://www.fil.ion.ucl.ac.uk/spm/) running on MATLAB 2019b. For this analysis, we included 174 subjects: (77 controls (53 women, median age 36 years (range 20–68)) and 97 patients (61 women, median age 39 years (range 18–76)), balanced for age (p = 0.08) and sex (p = 0.41). We have included detailed information about quality control and pre-processing in the “Functional connectivity” section of the Supplementary Material.

To investigate changes in the normal connectivity pattern of the DMN, we performed a study with the posterior cingulate cortex (P-cing, 0 − 51 21) as the seed. The time-series extracted from the region of interest (ROI) followed homogenisation procedures, excluding non-functionally representative voxels. The UF^2^C standard procedure excluded voxels with time-series that presented a low-outlier correlation with the seed-averaged time-series. We estimated the individual connectivity maps by using the Pearson correlation coefficient between the time-series from the P-cing seed and all times-series of grey matter voxels. We converted these maps to z-score maps by using with Fisher’s r-score to z-score transformation. We used the generalised linear model (GLM) from SPM12 to perform second-level analyses for group inferences. We compared patients and controls with a two-sample t-test controlling for sex and age. We also conducted separate linear regressions with individual maps and scores of fatigue (CFQ-11) and sleepiness (ESS), using sex and age as covariates. The results were corrected for multiple comparisons. We initially applied a statistical threshold of p < 0.001 (uncorrected at the voxel level, corresponding to a T > 3.16), with subsequent extent threshold at the cluster level with Benjamini–Hochberg procedure^52^ (false discovery rate [FDR] corrected at p < 0.05) to focus at the cluster level.

### Statistical analyses

We analysed the clinical data with SPSS 22. We used the chi-square test and Fisher’s exact test for categorical data. For continuous variables, we performed the non-parametric Kruskal–Wallis and Mann–Whitney tests. The R-software (http://www.R-project.org/org) was used for the analyses of neuropsychological variables^13^.

## Results

### Clinical characteristics

**Figure 1:**
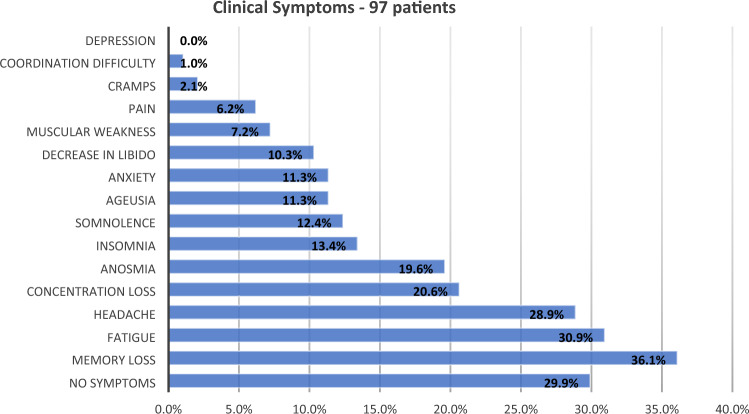
The graph shows the self-reported symptoms by 97 post-infected patients (median of 79 days post-infection).

We examined 97 unvaccinated individuals (61 women, median age 39 [range 18–76]), with a median interval between diagnosis and personal interview (and MRI acquisition) of 79 days and a mean of 97 days (range 30–420 days; SD = 71.7 days) (Table 1). The most frequent post-acute symptoms were memory difficulties (36.1%), fatigue (30.9%), headache (28.9%), and concentration difficulties (20.6%) (Fig. 1). Interestingly, fatigue was reported by 31 individuals (30.9%) and was mostly combined with other symptoms such as memory difficulties (17 subjects), headache (15 subjects) and concentration difficulties (10 subjects).

**Table 1:** Epidemiological Data; CFQ-11 and ESS were higher in the post-COVID patients, while education, age and sex were equivalent.

Data	Patients (97)			Controls (77)			p-value
	Mean	Median	Median CI 95%	Mean	Median	Median CI 95%	
Sex (female)	61/97 (62.9%)			53/77 (68.8%)			0.413
Age	41	39	[37.0–42.0]	38.2	36	[34.0–40.0]	0.082
Education (years)	15.7	16	[15.1–16.9]	16.9	16	[14.9–17.0]	0.192
CFQ-11 (Fatigue)	11.5	11	[9.2–12.8]	7.3	6	[4.3–7.7]	** < 0.001**
ESS (Daytime excessive somnolence)	8.6	8	[6.8–9.2]	6.9	6	[4.9–7.1]	**0.015**

Sex distribution was compared with Chi-square test; Continuous variables were compared with Mann–Whitney U tests.

*CFQ* Chalder fatigue questionnaire, *ESS* epworth sleepiness scale, *CI* confidence interval.

Significant values are in bold.

One radiologist (JGDS) visually inspected all the structural MRI scans and did not identify any significant alterations.

In addition to the semi-structured interview, the participants presented a median of 11 points (CI 95% 9.2–12.8) in the CFQ-11 and 8 points (CI 95% 6.8–9.2) in the ESS. Differently from the proportion of symptoms reported during the interview (fatigue in 30.9% and somnolence in 12.4%), the binary classification (presence or absence of symptoms) resulting from the scores showed symptoms of fatigue in 81 of 97 individuals (83%) and excessive daytime sleepiness in 34 of 97 individuals (35%).

The Pearson correlation coefficient between the CFQ-11 and ESS was moderate (r = 0.40, p < 0.001). Although excessive daytime sleepiness was more frequent in individuals with fatigue (32/81) compared to those without fatigue (2/16), the difference was not significant (p = 0.075).

### Neuropsychological evaluation

We performed the neuropsychological evaluation in a subset of 74 individuals (45 women, with a median age of 39 years [20–76]) as the remaining 23 subjects did not have time to complete the neuropsychological tests; no other reasons were reported.

**Figure 2:**
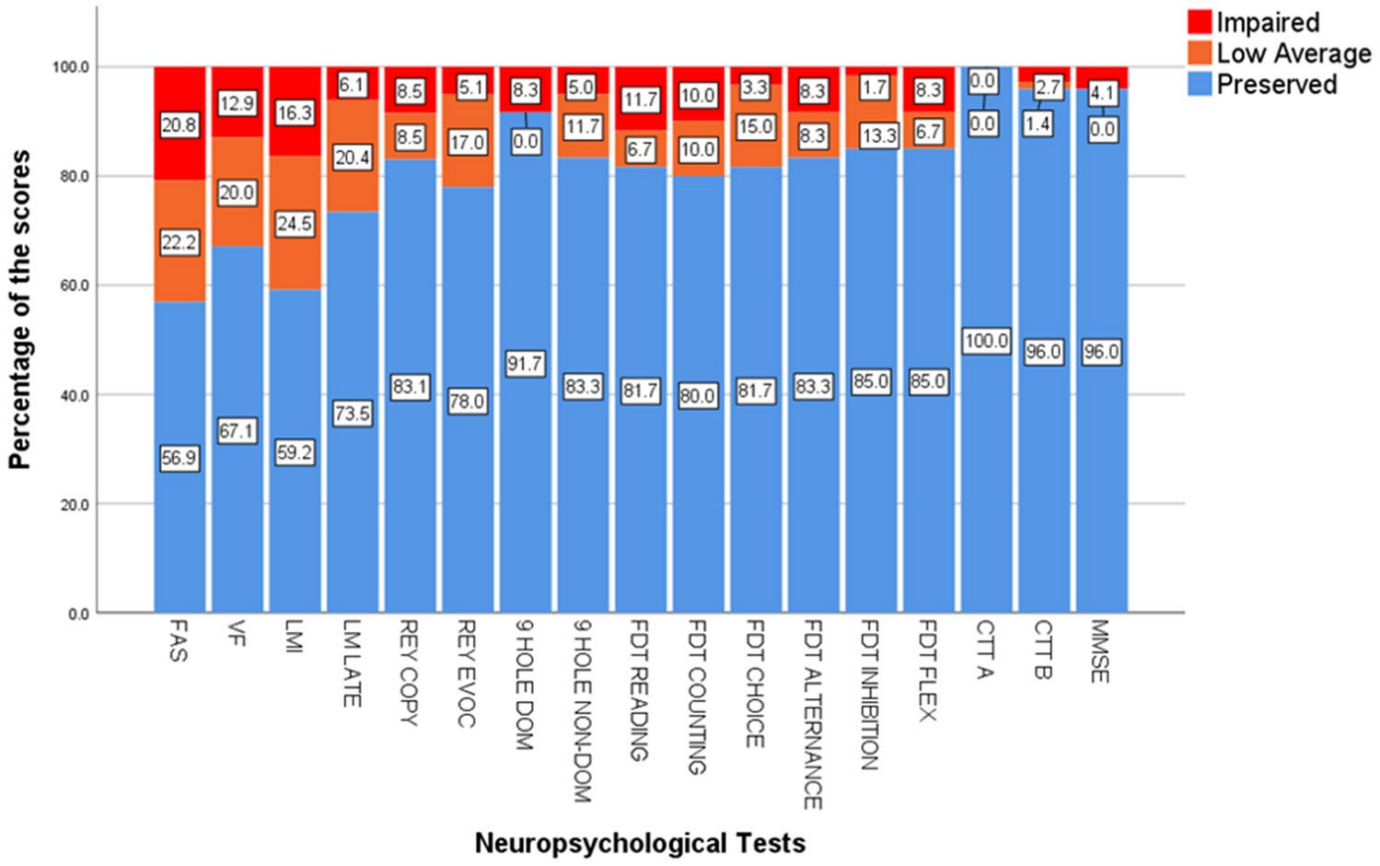
Neuropsychological evaluation of patients recovered from (mild) COVID-19 shows cognitive dysfunction mainly in in phonemic verbal fluency (FAS), verbal categorical fluency (VF) and logical memory tests (LM). *FAS* phonemic verbal fluency, *VF* verbal categorical fluency, *LMI* logical memory immediate recall, *LML* logical memory late recall, *9 Hole dom* 9-Hole Peg Test with dominant hand, *9-Hole non-dom* 9-Hole Peg Test with non-dominant hand, *FDT* five-digit test, *FDT* flex subtest of FDT for cognitive flexibility, *CTT* color trail test, *MMSE* mini-mental state exam.

In terms of cognitive performance, we identified abnormal performance (below low and exceptionally low scores) in 20.8% of patients for FAS, 16.3% in Logical Memory Immediate and 12.9% in verbal fluency (Fig. 2).

### Neuroimaging findings

#### DTI results

**Figure 3:**
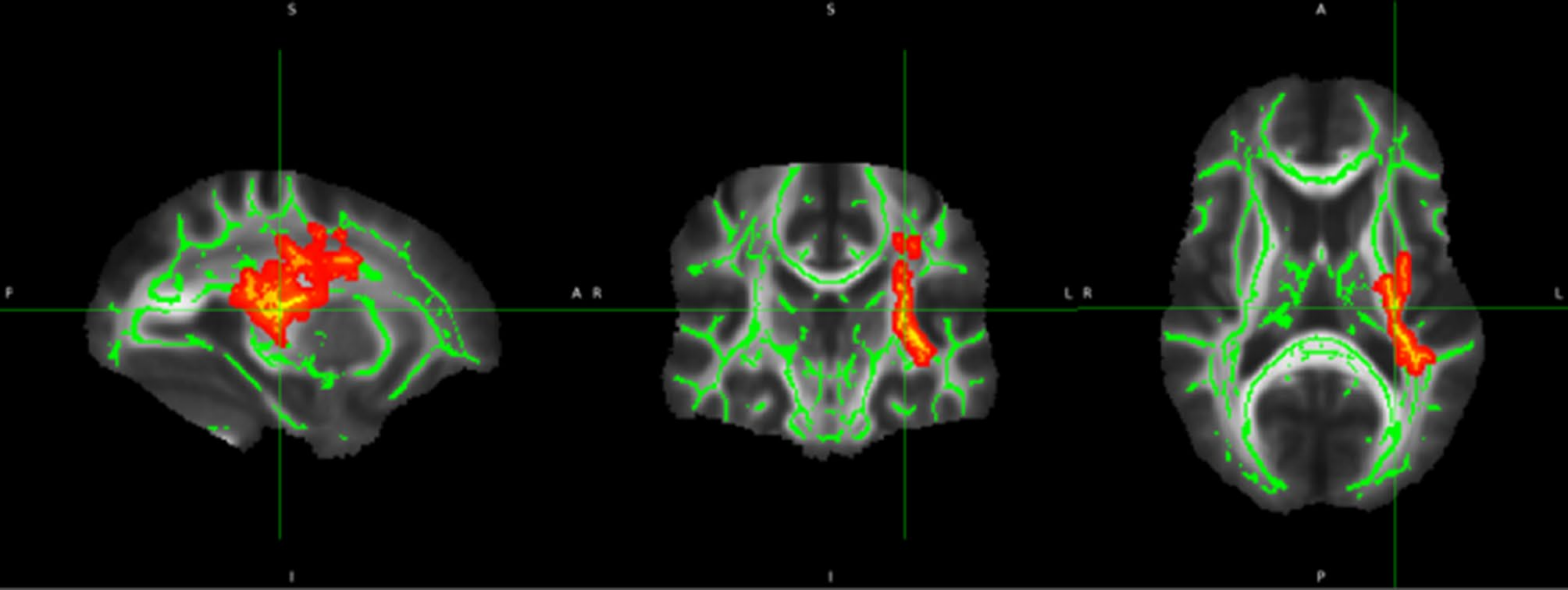
DTI results. Post-COVID patients presented higher axial diffusivity (red-yellow clusters) in the left hemisphere (results adjusted for multiple comparisons with Threshold Free Cluster Enhancement).

We analyzed 92 post-COVID patients and 77 controls balanced for age and sex. We did not find statistical differences in FA, MD and RD values. However, the patients presented higher AD values (Fig. 3, Table 2). Full statistical details are presented in the Supplementary Material.

**Table 2:** Clusters with increased axial diffusivity in patients (MNI coordinates in mm) compared to controls.

Voxels	p-value (TFCE-corrected)	p-min x (mm)	p-min y (mm)	p-min z (mm)	COG x (mm)	COG y (mm)	COG z (mm)	Anatomic region
1755	0.009	− 31	− 33	12	− 30.4	− 20.6	18.5	Left Anterior Thalamic radiation; CST; IFO; ILF; SLF
12	0.049	− 42	− 37	6	− 30.4	− 20.6	18.5	–
4	0.05	− 46	− 34	5	− 43.5	− 37.4	6.5	SLF
1	0.05	− 44	− 35	3	− 44	− 35	3	–

*SLF* superior longitudinal fasciculus, *CST* corticospinal tract, *IFO* inferior fronto-occipital fasciculus, *ILF* inferior longitudinal fasciculus.

The number of voxels inside the cluster (voxels), the significance value (p), the (x, y, z) coordinate with the highest significance value (p-min), and the (x, y, z) coordinates of the center of gravity (COG) of the cluster are provided. Results are presented with TFCE correction.

#### Correlations between DTI parameters and neuropsychological scores

There were no significant correlations between DTI parameters and neuropsychological scores.

#### FC of the DMN (seed-based on Pcing)

We compared 97 patients and 77 controls, paired for age (p = 0.082) and sex (p = 0.41), and we did not obtain significant differences after applying FDR correction.

Given the high proportion of subjects with symptoms of fatigue and sleepiness and previous associations between these symptoms and the DMN^19,20^, we further investigated the relationship between the DMN maps and scores of fatigue (CFQ-11 values; median 11, [CI95% 6.8–9.2])^43^; and sleepiness^53^ (ESS values, median 8, [CI 95%6.8–9.2])^45^. We performed two separate linear regressions between the FC maps and the ESS and CFQ-11. No significant results were observed.

## Discussion

We evaluated a group of 97 unvaccinated individuals (without history or current presence of symptoms of anxiety, depression and psychiatric symptoms) after a mild infection with SARS-CoV-2. We detected persistent headache, fatigue, excessive somnolence, cognitive dysfunction, and subtle microstructural white MRI abnormalities. Although the DTI analysis revealed an increase in axial diffusivity compared to controls, the investigation of the Default Mode Network with resting-state fMRI did not differ between patients and controls. In addition, the intensity of fatigue and somnolence did not correlate with the functional connectivity of the DMN.

### Persistent clinical symptoms

The proportion of female responders to our online questionnaire for recruitment was higher than male responders, comparable to another study^54^ that conducted an online survey. There is as yet no clear evidence if sex is a risk factor for developing or perpetuating post-infectious effects of COVID-19. However, a recent meta-analysis of 134 cohorts reported that women and female cohorts were more likely to have experience deterioration of mental health^55^. Besides, other studies have demonstrated that female gender is associated with long COVID syndrome^56^ and more frequent post-infection fatigue^44^.

Two months after the acute period of COVID-19 infection, the most frequently reported symptoms were memory difficulties (36.1%), fatigue (30.9%), and headache (28.9%), following previous reports^54,57^. Our findings are in accordance with one recent study that compared 138,818 individuals with COVID-19 infection with 5,985,227 without COVID-19 after 2 years of follow-up and confirmed an increased risk of post-acute sequelae in non-hospitalized individuals, including mental health problems, neurologic dysfunction, and fatigue^58^. Although fatigue was self-reported by one-third of patients, the examination of CFQ-11 scores revealed a higher frequency of fatigue in patients (83.5%) compared to controls (62%), confirming that symptoms of fatigue persist regardless the severity of acute infection, as previously reported in a group of 128 individuals examined with a median interval of 10 weeks after the initial symptoms of COVID-19^44^. As recently reported^59^, we also identified excessive somnolence in the group of patients (35%), compared to the controls (11%). Overall, after excluding subjects with current (or past) symptoms of anxiety (or depression), we demonstrate persistent symptoms in a group of mildly infected, highly educated individuals.

### Cognitive dysfunction and COVID-19

Recent studies have confirmed post-infectious cognitive dysfunction in survivors of COVID-19 (from ICU and ward hospitalization), and in non-hospitalized subjects^3,9,10,60^. However, cognitive impairment after mild infection (without hospitalization) is not well understood. In addition, it is not clear how hospitalization impacts neuropsychological functions, as literature is inconsistent in showing neuropsychological differences related to acute phase severity or hospitalization^56,61,62^. In a recent analysis of 35 patients after hospital discharge (12.6 years of education and approximately 26 days after discharge), the application of supervised neuropsychological tests revealed abnormal performances mainly in verbal fluency (11.4%), mental flexibility and working memory (8.6%)^60^. Our group of 74 non-hospitalized participants presented higher rates of impairments in phonemic verbal fluency (20.8%), semantic verbal fluency (16.3%), episodic immediate verbal memory (16.3%), and processing speed (11.7% in FDT-Reading and 10% in FDT-Counting). Compared with the above-mentioned study, we examine more participants and detected cognitive impairment, after a longer interval since the acute infection (approximately 79 days). So far, other researchers have applied a variety of non-supervised cognitive tests^3,63^ and also have demonstrated attention deficits in survivors. Despite the methodological differences (use of “web-optimized assessment”), one study^63^ evaluated a large sample of 81,337 individuals (although only 326 subjects had a confirmed COVID-19 diagnosis) and detected abnormalities in “higher cognitive or executive function”, especially in tasks with a semantic component, and in those requiring selective visual attention.

In a study^64^ that evaluated the cognitive functions of 740 patients (379 non-hospitalized; approximately 7.6 months after the COVID-19 infection), impairments were observed in processing speed attention, executive functions, phonological and semantic verbal fluency, and associated deficits in memory considering the coding and evocation steps. Interestingly, if we apply − 1.5 z-score as the criteria for impairment, we observe impaired phonemic verbal fluency in 16% of our group, which is comparable to the 11% reported. In addition, features of dysexecutive syndromes, including confusion and attentional difficulties, have been associated with cognitive complaints presented by patients after COVID-19 infection^65^. The online evaluation of a group of 18 young post-infected subjects (mean age 42 years) with median interval of 85 days after diagnosis detected alterations of short-term memory, attention and concentration^66^.

Guo et al.^67^ assessed cognitive deficits with online evaluation (including memory, language, and executive functions). The study compared 181 patients with self-reported COVID-19 (only 65 confirmed, combining mild, moderate and severe acute infections) with 185 healthy controls and reported poor memory performance when compared to the control group.

A systematic review and metanalysis^10^ analyzed 175 patients who recovered from COVID-19 and confirmed cognitive dysfunction compared to healthy controls. Despite the methodological differences (related to cognitive assessments, the inclusion of patients with severe acute infection and varied intervals after acute infection), which may have contributed to the moderate level of heterogeneity among studies (I^2^ = 63%), the sensitivity analysis yielded similar outcomes—suggesting the persistence of cognitive impairment, at least from 1 to 6 months.

Unfortunately, methodological variabilities (considering the inclusion criteria, interval after the diagnosis, and the severity of acute infection and assessment methods) among these cognitive studies compromise comparisons. However, regardless of these methodological differences (and variations in the interval after diagnosis), we also observed dysfunctions in language and executive functions, episodic memory, and processing speed that agree with the literature data. The fact that we detected cognitive impairment almost one year after the diagnosis for some individuals is not surprising, as one recent study confirmed the risk of cognitive dysfunction after 2 years^9^.

One intriguing fact is that we observed a high proportion of low average performance in our sample of patients (which has a high average level of education), including immediate and late verbal episodic memory, phonological and semantic verbal fluency, immediate visuospatial episodic memory, processing speed, and inhibitory control. Although most subjects did not present significant impaired scores compared with the normative data, we speculate that the low average performance affecting different domains may result in a negative impact in everyday life, especially in individuals with high levels of education and cognitive demands.

### MRI analyses

#### DTI alterations

Although contradicting, some studies have demonstrated changes in WM in hospitalized survivors^14,15,23,68^. However, fewer investigated WM alterations in individuals after a mild infection^16^. While the evaluation of 22 hospitalized patients with 1-year follow-up showed lower FA in the corpus callosum of severe patients, another study with 60 hospitalized patients (3 months after discharge) observed higher FA associated with reduced MD, RD and AD^14^. Likewise, high fractional anisotropy was reported in the study of one individual with a post-COVID autoimmune encephalitis^69^. We recently evaluated 56 patients with mild-to-moderate acute infection (95% non-hospitalized, without assessment of symptoms of anxiety or depression)^16^ and did not identify significant alterations of FA, AD, RD and MD; however, there was a significant reduction of fiber density in different tracts, mostly in the left hemisphere. The analyses of 86 patients (29 hospitalized and 57 non-hospitalized; the average age of 50 years and approximately one year after the acute infection) compared to 36 controls identified an overall reduction of fractional anisotropy, mean diffusivity, axial diffusivity and radial diffusivity^70^. Contrary to our group of non-hospitalized subjects (without history or current neuropsychiatric symptoms), the authors included individuals with hospitalization (33%), symptoms of depression (27%; mean BDI-II of 14 points), anxiety (9%), and older age (mean age of 50 years). We raised the hypothesis that the reduction of DTI measurements in the study of Diez-Cirarda et al.^70^ (contrary to the elevated values of axial diffusivity in the present study) and the absence of changes in the study performed by Bispo et al.^16^ could be associated with the combination of different methodology and the clinical differences of the groups included in each study.

We considered the absence of past history or current presence of neuropsychiatric symptoms in our group of patients necessary to disentangle the interaction between symptoms of anxiety (and depression) and the COVID infection, considering the possible negative impact of both on the white matter structure. We had previously reported an association between intensity of anxiety symptoms and atrophy of orbitofrontal cortex^13^; another study of 42 patients (37 hospitalized) identified a negative correlation between BDI scores with AD in a small cluster in the left hemisphere^23^. Therefore, our present results suggest a specific impact of the SARS-CoV-2 in the white matter independent of mood changes. However, the significance and interpretation of higher values of AD in the absence of alterations of other diffusivities requires extreme caution, due to the complexity of biophysical properties of these metrics^71^. As we did not identify significant correlations between maps of AD and scores of fatigue, somnolence or cognitive tests, we speculate the possibility of a silent biological process driven by the infection (direct viral or immune activation) which we were unable to associate with any cerebral dysfunction. As increases of axial and radial diffusivities have been associated with aging in healthy adults^72^, the longitudinal analyses may elucidate if the increase of AD will persist and eventually reflect any accelerated aging of the white matter.

#### FC analyses

While some studies reported alterations of brain functional connectivity after acute COVID-19^22,41,70,73,74^, we did not identify differences of functional connectivity between groups of patients and controls. Nor did we observe correlations of Pcing connectivity with fatigue or somnolence. Fewer studies reported Default Mode Network alterations after COVID-19^74,75^. One group of researchers described higher intranetwork connectivity within the DMN and higher internetwork connectivity between the DMN and the Olfactory Network (13 controls and 22 subjects with olfactory dysfunction without information related to the severity of infection)^74^. Another study performed functional connectivity analysis (with whole brain, region-of-interest based technique) of a group with 45 subjects after COVID-19 (17 mild, 19 moderate, nine severe, without healthy controls) and reported a role of the Default Mode Network in neuropsychiatric alterations^75^.

Some reasons (other than the different methods applied to analyze functional connectivity)^22,41,70,73,74^ may explain the discrepancies between our negative findings and different patterns of alterations detected in the previous analyses. One major factor is the inconsistencies related to the definition of post-COVID syndrome, which has been heavily criticized recently^26^. Although some studies included patients after 3 to 6 months after acute infection^41,73,76^, one study evaluated hospitalized patients 2 weeks after the acute infection^22^. The severity of the initial infection is another critical factor for neuropsychological evaluation and neuroimaging analyses (as severe infection associated with long-term impaired cognition^77,78^ and cerebral atrophy, regardless the etiology^29^; however two studies exclusively evaluated hospitalized patients^22,76^, one did not specify^74^, and the other two studies (from the same group) compared patients with mild (21 subjects), moderate (20 subjects) and severe infection (9 subjects). Unfortunately, these last two studies^41,73^ only included a small group of mildly infected individuals (21 subjects) and did not include healthy controls to determine the true deviations from what would be considered as the normal pattern (compared to paired non-infected individuals). The presence of symptoms of anxiety and depression (which may affect functional connectivity regardless of the presence of systemic infection^79^ was not clearly described in most of the studies^22,41,74^. One study with 50 hospitalized patients demonstrated increased functional connectivity associated with severity of post-traumatic stress symptoms^76^, considering that these individuals also presented symptoms of anxiety and depression. Given all the differences between these previous reports and our group, we were not surprised with the absence of alterations of cerebral functional connectivity since our group of mildly infected patients did not present a history or current symptoms of anxiety (or depression), and was compared to a balanced group of non-infected individuals (who underwent the same stressful conditions of COVID pandemics).

## Limitations

The nature of this study—a cross-sectional design with a convenience sample—has restrained our ability to generalise our findings as more symptomatic individuals (mainly women) may have enrolled. Unfortunately, we cannot exclude a previous asymptomatic SARS-CoV2 infection for the controls enrolled after the vaccination campaign—we could not test them as their serologies could be positive due to the vaccines or an unnoticed earlier infection. It is noteworthy that part of our sample may not fulfil other definitions of post-COVID condition (or post-acute sequelae of SARS-CoV-2 infection, or long COVID) such as WHO or NICE criteria^24^. However, our initial observations raise concerns about possible long-term impairment in patients recovered from mild COVID-19 (even though some subjects may not be aware of their losses or dysfunction), especially considering the limited understanding of the neurological impact of SARS-CoV-2 infection. Nevertheless, our longitudinal analyses with a larger sample will provide additional insight regarding these long-term, persistent symptoms related to cognition and brain connectivity.

## Conclusions

Our findings suggest that SARS-CoV-2 affects the brain in individuals who did not require hospitalization. Some subjects present persistent fatigue, headache, memory problems, and somnolence even 2 months after their COVID-19 diagnosis. We detected cognitive impairment in these individuals, along with subtle white matter abnormalities. The subtle brain alterations and the presence of cognitive dysfunction (in the absence of symptoms of anxiety and depression) raise the need for longitudinal follow-up of patients recovered from COVID-19, even in those mildly infected. Specific treatment of symptoms and neurorehabilitation strategies may be necessary to improve the quality of life and cognitive function for those with persistent limitations after the acute phase.

### Supplementary Information

Supplementary Information.

## Data Availability

Data collected and analysed for this study will be available in the University of Campinas database. The raw neuroimaging data will be available upon reasonable request to Dr Clarissa Yasuda.
