# Supplementary material for: Microstructural brain abnormalities, fatigue, and cognitive dysfunction after mild COVID-19
Source: Sci Rep. 2024 Jan 19;14:1758. doi: 10.1038/s41598-024-52005-7 (PMC10798999; doi:10.1038/s41598-024-52005-7)
Supplement: Supplementary file 1 — Supplementary Information. [file 41598_2024_52005_MOESM1_ESM.docx]

**Microstructural brain abnormalities, fatigue, and cognitive dysfunction after mild COVID-19**

Lucas Scardua-Silva#^1,2^, Beatriz Amorim da Costa#^1,2^, Ítalo Karmann Aventurato#^1,2^, Rafael Batista Joao^1,2^, Brunno Machado de Campos^1^, José Flávio Bechelli^1,2^, Leila Camila Santos Silva^1,2^, Alan Ferreira dos Santos^1,2^, Mariana Rabelo de Brito^1,2^, Marina Koutsodontis Machado Alvim^1,2^, Guilherme Vieira Nunes Ludwig^1,3^, Cristiane Rocha^1,4^, Thierry Kaue Alves Silva Souza^1,2^, Maria Julia Mendes^1,2^, Takeshi Waku^1^, Vinicius de Oliveira Boldrini^1,5^, Natália Silva Brunetti ^5^, Sophia Nora Baptista^5^, Gabriel da Silva Schmitt^2^, Jhulia Gabriela Duarte de Sousa^6^, Tânia Aparecida Marchiori de Oliveira Cardoso^2^, André Schwambach Vieira^4,5^, Leonilda Maria Barbosa Santos^5^ , Alessandro dos Santos Farias^5^, Mateus Henrique Nogueira*^1,2^, Fernando Cendes*^1,2^, Clarissa Lin Yasuda*^1,2^

**Supplementary File**

**Material and Methods**

| **Supplementary Figure 1.** Description of exclusions of participants due to clinical and technical issues |
| --- |
| **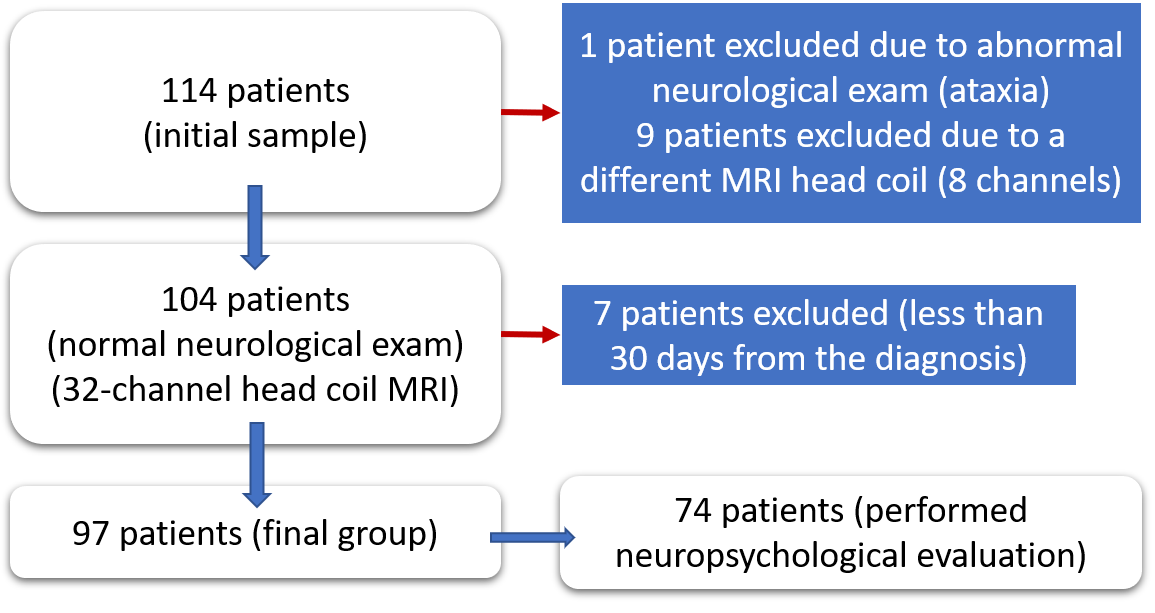** |

Participants

We included 114 consecutive participants (without history or current symptoms of anxiety or depression) in our initial sample (Supplementary figure 1). One patient was excluded due to post-COVID ataxia (neurological examination) and nine because their MRI was performed with the 8-channel head coil. Seven participants presented less than 30 days from their infection by SARS-CoV-2. From our 97 remaining patients, 74 patients performed the neuropsychological tests.

Neuropsychological Instruments

• **Mini-Mental State Examination (MMSE)**: the MMSE is a widely used instrument for screening cognitive function in general. It is an easy-to-apply instrument, lasting approximately 10 minutes. The MMSE evaluates some cognitive domains, such as spatial and temporal orientation, immediate episodic memory and evocation, calculation, language, and naming, in addition to visuoconstruction and perception^1^.

Semantic **Verbal Fluency Test (FVS)^2,3^**: evaluates the semantic elocution in one minute of words belonging to the animal category. This category is substantially used in cognitive assessments as it is sensitive to organization screening and semantic mental lexical access.

**Phonemic Verbal Fluency Test (FAS)^3^**: the F-A-S Verbal Phonemic Fluency Test is widely mentioned in the scientific literature. It uses the most frequent letters in Brazilian Portuguese, similar in sensitivity to the Phonemic Fluency Test used in other languages. The evocation time of each letter is one minute, totaling three minutes of application.

**Logical Memory subtest** from the Wechsler Memory Scale (WMS-R)^4,5^: the logical memory subtest is used in the clinical and scientific research contexts to assess verbal episodic memory (immediate and late recall). This subtest contains two stories with 25 items subdivided into thematic contents grouped into character characterization, conflict, development of the story, and conclusion. Patients are asked to replay the story immediately after reading it and again after a 30-minute break.

**Rey Complex Figure (A):** the Rey Complex Figure investigates processes of visuospatial construction, cognitive flexibility, planning, speed and precision, and immediate and delayed visuospatial episodic memory. The visual stimulus consists of a complex geometric figure formed by several interconnecting elements that must be copied and reproduced three minutes after the initial copy^6^.

**9-Hole Peg Test:** this instrument measures manual dexterity and fine motor coordination. The task consists of placing a pin in each of the nine holes in a base and removing them in the shortest possible time. The researcher clocks the time, which is the performance measure for both the dominant and non-dominant hand^7^ .

**Five Digit Test** (FDT): the FDT investigates aspects of attention and executive functions, including cognitive processing speed, the ability to focus and refocus attention, and the ability to deal with interference. The first part of the test assesses automatic cognitive processes, including the reading and counting subtests. The second part of the FDT requires controlled actions that mobilize superior mental resources, including selective attention, cognitive flexibility, and inhibitory control^8^.

**Color Trails Test**: this test is an adaptation with Brazilian standardization of the internationally known Color Trails Test (CTT) that aims to evaluate the attentional processes, involving aspects of sustained and divided attention. It is divided into two stages, the first of which aims to evaluate processes of automation and sustained attention. In the second stage, alternated attention is evaluated, in addition to cognitive flexibility^9^.

The **Beck Depression Inventory (BDI-II)^10^** is a self-administered scale used for screening the severity of depressive symptoms. It consists of a standard multiple-choice questionnaire with 21 questions containing four items each. The total score ranges from 0 to 63, and the levels of severity thresholds follow 0-13 for minimal or no depression; 14-19 for mild depression; 20-28 for moderate depression; and 29-63 for severe depression symptoms.

The **Beck Anxiety Inventory (BAI)^11^** is also a self-administered scale for screening the severity of anxiety symptoms. This scale consists of 21 items with descriptive statements of physical and subjective symptoms of anxiety. The total score ranges from 0 to 63, allowing the characterization of intensity levels according to the following cutoffs: 0-10 for minimal or asymptomatic anxiety symptoms; 11-19 for mild anxiety; 20-30 for moderate anxiety; and 31-63 as severe anxiety symptoms.

The **Chalder Fatigue Scale** (CFQ-11)^12^ questionnaire was used to assess the severity of fatigue. The participants answer 11 items on a 4-point scale (0-3). The global score varies from 0-33. The scale can be used to separate “cases” and “non-cases” based on a binary fatigue score ranging from 0-11. According to the binary system, scores of 4 or more are considered “cases” with fatigue^13^.

We used the **Epworth Sleepiness Scale (ESS)** to estimate excessive daytime sleepiness (EDS). It is a self-report questionnaire with eight situations involving daily activities. The final global scores range from 0-24; the diagnosis of EDS is suggested for those with scores higher than 10^14^.

Assessment of individual complaints: during the face-to-face interview, we asked how the subject had been feeling in the last week. They could choose from a list of symptoms (headache, smell alteration, taste alteration, tiredness or fatigue, drowsiness during the day, memory problems, difficulty concentrating, difficulty with daily activities, motor difficulties, seizures, fainting, pain, muscle weakness, cramps, "I have no complaints, and I am fully recovered", anxiety, depression, insomnia, or sleeping difficulty, decreased sexual libido) and include other answers.

MRI Protocol (3T Philips-Achieva) designed for the NEUROCOVID study:

- Axial diffusion-weighted image (DWI) with acquisition voxel sizes of 1.51x1.95x3mm³ reconstructed with 0.9x0.9x3mm³, 55 slices, gap=0.3mm, TR=3776ms, TE=94ms, flip angle=90º and FOV=230x230mm².
- Axial susceptibility weighted image (SWI) with acquisition voxel sizes of 0.6x0.6x2mm³ reconstructed with 0.34x0.34x1mm³, 142 slices, no gap, TR=42ms, 6 echoes, first echo=7.19ms and echospacing=6.2ms, flip angle=17º and FOV=230x189mm².
- Sagittal T1 3D WI with isotropic voxels of 1mm, acquired in the sagittal plane, 1mm thick, no gap, flip angle=8°, TR=7.0ms, TE=3.2ms, matrix=240x240, FOV=240x240mm^2^
- Sagittal T2-3D fluid-attenuated inversion recovery image (FLAIR) with isotropic voxels of 1.2mm, reconstructed with 0.5x0.5x0.6mm³, 300 slices, gap=0.6mm, TR=10000ms, TI=2680ms, TE=276ms, 2 averages (sampling averages), FOV=250x250mm^2^
- Resting-state: Echo planar images (EPI) with voxel sizes of 3x3x3 mm³, acquired on the axial plane with 40 slices, no gap, flip angle=90º, TR=2s, TE=30ms in a 6-minute scan resulting in 180 dynamics and FOV=240x240mm².
- DWI/DTI: Diffusion tensor image (multiple diffusion direction images) with acquisition voxel sizes of 2x2x2mm³ reconstructed with 1x1x2mm³, 70 slices, no gap, TR=8500ms, TE=61ms, flip angle 90°; 32 gradient directions; no averages; max b-factor=1000s/mm², FOV=256x256mm².

Functional connectivity

We performed the functional connectivity study using the UF²C toolbox (https://www.lniunicamp.com/uf2c) within SPM12 (http://www.fil.ion.ucl.ac.uk/spm/, using MATLAB 2019b) ^15^. These images were preprocessed and analyzed with the UF²C standard pipeline. The preprocessing was based on functional image realignment, normalization to the MNI-space (Montreal Neurologic Institute Standard template), co-registration with T1W image, framewise displacement (FD), the derivative variance (DVAR) estimation (head movement parameters) and smoothing with a kernel of 6x6x6 mm (FWHM). Additionally, the structural images were segmented, modulated, and normalized (to the MNI space). After the initial preprocessing, the functional images were detrended (to remove MR linear trend), band-pass filtered (0.008-0.1Hz), and regressed for censoring vectors (framewise displacement (FD)>0.5mm), white matter (WM), and CSF average signal, and for six realignment parameters.

Diffusion data analysis with TBSS

*Pre-processing*

We used the recommended pipeline from the ENIGMA consortium (https://enigma.ini.usc.edu/) to pre-process DTI data. Each subject`s raw data were initially denoised using the Marchenko-Pastur PCA algorithm implemented in the MRtrix3 toolbox (https://www.mrtrix.org/)^16-19^ followed by Gibbs ring artifact removal^19,20^. A synthetic B0 image was produced using the SynB0-DisCo algorithm^21^ and used alongside FSL’s “*topup”* and “*eddy* commands” (https://fsl.fmrib.ox.ac.uk/fsl/fslwiki/) for movement and concurrent eddy current correction with slice-to-volume movement model and susceptibility-by-movement correction^22-25^. The resulting image was further processed using the ANTS bias field correction algorithm^26^ before fitting the tensor model using FSL’s “*dtifit”* to generate fractional anisotropy (FA), axial diffusivity (AD), mean diffusivity (MD) and radial diffusivity (RD) maps.

*Voxel-wise analysis*

Following the TBSS pipeline (https://fsl.fmrib.ox.ac.uk/fsl/fslwiki/TBSS) ^27^, FA images from all subjects were normalized to the FMRIB58_FA standard space and averaged to produce a mean FA image. The mean image was skeletonized using the TBSS algorithm. The mean FA skeleton was thresholded at 0.3 by visual inspection in order to exclude juxtacortical white matter tracts from the final skeleton. Finally, subject FA data were projected into the mean FA skeleton mask to produce subject-specific skeletonized FA maps. We used the skeletonized FA maps for the final voxel-wise statistical analysis. Groups were compared using a GLM controlling for age and sex. Multiple comparison correction was performed using Threshold Free Cluster Enhancement^28^ as implemented in FSL’s *randomize^29^* with parameters adjusted for the skeletonized data (H=2, E=1, 26-voxel-connectivity) and a null distribution estimated using 10,000 random permutations of the data.

Additional GLMs were produced testing the correlation of FA maps with phonetic fluency, semantic fluency, LM (immediate recall) and LM (late recall) z-scores in post-COVID subjects as well as CFQ-11 and ESS scores in both post-COVID subjects and controls. Similarly, AD, MD and RD maps were transformed to standard space and projected into the mean FA skeleton mask for statistical using the same techniques described above.

*Description of results*

We used the “*atlasquery*” FSL’s tool (with the JHU White-Matter Tractography Atlas) to describe the results from the statistical analysis.

Supplementary results

S**upplementary Table 1**. Detailed description of Neuropsychological Data

| Variable | n | Missing | Minimum | Maximum | **Mean** | CI95% mean - lower limit | CI95% mean - upper limit | **Median** | Asymptotic CI95% median - lower limit | Asymptotic CI95% median - upper limit |
| --- | --- | --- | --- | --- | --- | --- | --- | --- | --- | --- |
| FAS Total | 72 | 13% | 15.00 | 72.00 | 36.39 | 33.65 | 39.13 | 35.00 | 31.62 | 38.38 |
| FAS z score | 72 | 13% | -2.94 | 3.37 | -0.42 | -0.69 | -0.14 | -0.48 | -0.82 | -0.14 |
| VF (animals) – total | 71 | 14% | 8.00 | 34.00 | 19.87 | 18.68 | 21.06 | 20.00 | 18.53 | 21.47 |
| VF - z score | 71 | 14% | -2.54 | 2.74 | 0.01 | -0.27 | 0.29 | -0.09 | -0.43 | 0.26 |
| LMI - total | 49 | 41% | 8.00 | 33.00 | 21.98 | 20.14 | 23.82 | 22.00 | 19.75 | 24.25 |
| LMI z score | 49 | 41% | -2.25 | 1.08 | -0.40 | -0.65 | -0.15 | -0.50 | -0.81 | -0.19 |
| LML - total | 49 | 41% | 7.00 | 33.00 | 19.20 | 17.38 | 21.03 | 20.00 | 17.77 | 22.23 |
| LML - z score | 49 | 41% | -1.62 | 1.88 | -0.20 | -0.42 | 0.02 | -0.14 | -0.41 | 0.12 |
| Rey Complex Figure – Copy | 59 | 29% | 14.50 | 36.00 | 33.65 | 32.50 | 34.81 | 36.00 | 34.58 | 37.42 |
| Rey Complex Figure – Copy z-score | 59 | 29% | -4.67 | 1.09 | 0.39 | 0.09 | 0.69 | 0.99 | 0.62 | 1.36 |
| Rey Complex Figure - Evocation | 59 | 29% | 1.50 | 32.50 | 19.09 | 17.22 | 20.97 | 20.00 | 17.70 | 22.30 |
| Rey Complex Figure – Evocation z-score | 59 | 29% | -2.60 | 2.08 | 0.32 | 0.03 | 0.61 | 0.35 | -0.01 | 0.70 |
| 9 Hole Mean dominant hand | 60 | 28% | 15.00 | 28.50 | 20.32 | 19.46 | 21.17 | 19.75 | 18.70 | 20.80 |
| 9 Hole Mean dominant – z-score | 60 | 28% | -3.07 | 2.11 | 0.48 | 0.24 | 0.72 | 0.73 | 0.43 | 1.03 |
| 9 Hole Mean non-dominant hand | 60 | 28% | 15.00 | 30.00 | 20.88 | 20.06 | 21.69 | 20.75 | 19.75 | 21.75 |
| 9 Hole Mean non-dominant – z-score | 60 | 28% | -2.99 | 2.18 | 0.32 | 0.07 | 0.56 | 0.49 | 0.19 | 0.78 |
| FDT Reading | 60 | 28% | 14.00 | 47.00 | 23.93 | 22.18 | 25.69 | 22.00 | 19.85 | 24.15 |
| FDT Reading z-score | 60 | 28% | -4.46 | 1.43 | -0.03 | -0.32 | 0.26 | 0.29 | -0.06 | 0.65 |
| FDT Counting | 60 | 28% | 18.00 | 50.00 | 27.27 | 25.56 | 28.98 | 26.00 | 23.90 | 28.10 |
| FDT Counting – z-scores | 60 | 28% | -3.31 | 1.31 | -0.09 | -0.35 | 0.18 | 0.21 | -0.11 | 0.54 |
| FDT Choice | 60 | 28% | 23.00 | 90.00 | 40.62 | 37.94 | 43.30 | 39.00 | 35.71 | 42.29 |
| FDT Choice – z-score | 60 | 28% | -3.33 | 1.39 | 0.03 | -0.16 | 0.23 | 0.20 | -0.05 | 0.44 |
| FDT Alternation | 60 | 28% | 30.00 | 107.00 | 52.57 | 48.64 | 56.49 | 50.50 | 45.68 | 55.32 |
| FDT Alternation – z-score | 60 | 28% | -2.90 | 1.23 | 0.01 | -0.20 | 0.22 | 0.17 | -0.10 | 0.43 |
| FDT Inhibition | 60 | 28% | -1.00 | 62.00 | 16.68 | 14.26 | 19.11 | 16.50 | 13.52 | 19.48 |
| FDT Inhibition – z-score | 60 | 28% | -3.68 | 1.66 | 0.06 | -0.15 | 0.28 | 0.15 | -0.11 | 0.41 |
| FDT Flexibility | 60 | 28% | 6.00 | 79.00 | 28.63 | 25.12 | 32.14 | 26.00 | 21.69 | 30.31 |
| FDT Flexibility – z-score | 60 | 28% | -3.14 | 1.51 | 0.03 | -0.20 | 0.26 | 0.10 | -0.19 | 0.38 |
| CTT 1 – time | 74 | 11% | 21.00 | 109.00 | 38.09 | 34.82 | 41.37 | 34.00 | 29.96 | 38.04 |
| CTT 1 - z-score | 74 | 11% | -0.46 | 1.41 | 0.67 | 0.58 | 0.75 | 0.74 | 0.64 | 0.85 |
| CTT 2 – time | 74 | 11% | 44.00 | 306.00 | 87.23 | 78.05 | 96.41 | 75.50 | 64.19 | 86.81 |
| CTT 2 – z-score | 74 | 11% | -1.35 | 1.39 | 0.51 | 0.39 | 0.63 | 0.62 | 0.47 | 0.76 |
| Total MEEM | 74 | 11% | 23.00 | 30.00 | 29.15 | 28.86 | 29.43 | 30.00 | 29.65 | 30.35 |
| MMSE – z-score | 74 | 11% | -2.36 | 1.46 | 0.93 | 0.75 | 1.10 | 0.99 | 0.77 | 1.21 |

FAS: Phonemic Verbal Fluency Test; VF: Semantic Verbal Fluency Test; LMI: Logical Memory (immediate recall); LML: Logical Memory (late recall); 9 Hole: 9 Hole Peg Test; FDT: Five Digit Test; CTT: Color Trails Test; MMSE: Minimental State Exam;

**Supplementary Table 2. Online questionnaire - SECTION 1 / 2**

| We are researchers from the Department of Neurology (School of Medical Sciences / UNICAMP) and from the Department of Biology (UNICAMP), and we are studying the effects of coronavirus in the central nervous system.  This questionnaire will help us to understand how people are recovering themselves after the infection by the new coronavirus. Our complete project includes a magnetic resonance, neurological and cognitive examination (memory, language…). If it is possible for you to answer this questionnaire, we would be very grateful.  Principal investigator: Prof. Clarissa Lin Yasuda (CRM 94104)  Contact: [neurocovid@hc.unicamp.br](mailto:neurocovid@hc.unicamp.br) (19) 99768-7517  *****Required | | | | | | | |
| --- | --- | --- | --- | --- | --- | --- | --- |
| **E-mail address*** | (Your e-mail) | | | | | | |
| **Name*** | (Your answer) | | | | | | |
| **Age*** | (Your answer) | | | | | | |
| **Gender** | Male | Female | | I prefer not to answer | | Other: | |
| **Phone** | (Your answer) | | | | | | |
| **City / State** | (Your answer) | | | | | | |
| **Do you have any association with UNICAMP?**  (Are you a student, professor or worker at UNICAMP?) | Yes | No | | | | | |
| **Date of diagnosis** (COVID infection)* | Date of confirmation (with a test) | | | | | | |
| **Diagnostic Method**  What was the confirmation’s method of the Covid diagnosis? | PCR (Swab) | | Antibodies (Blood test / Quick test) | | Antibody + PCR | | Other: |
| **Treatment**  Select the type of treatment for the COVID infection | Home | | Hospital - Infirmary | | Hospital – Intensive Care Unit | | |
| **Symptoms of acute infection***  Describe the symptoms you presented during the acute period (in the hospital or individual isolation, in case you had the treatment done). Select all the relevant symptoms. | Shortness of breath | | Tiredness / Fatigue | | Fever | | |
|  | Olfactory changes | | Headache | | Taste changes | | |
|  | No symptoms | | Other: _______________________________ | | | | |
| **Symptoms in the first month after hospital discharge / quarantine**  Describe the symptoms in the period after the hospital discharge or after the end of individual isolation (If you had your treatment at home). Select all the relevant symptoms. | Shortness of breath | | Tiredness / Fatigue | | Fever | | |
|  | Olfactory changes | | Headache | | Taste changes | | |
|  | No symptoms | | Other: | |  | | |

After section 1, continue to section 2

**SECTION 2 / 2**

| **SYMPTOMS AFTER COVID INFECTION**  In this section, we will talk about the symptoms that occurred after the recovering of COVID-19 infection. | | | | | |
| --- | --- | --- | --- | --- | --- |
|  | | | | | |
| **Frequent Post-Covid Symptoms**  Check the symptoms that had after the infection, but may not necessarily persisted up to now. | Headache | | Olfactory changes | | Taste changes |
|  | Tiredness / Fatigue | | Shortness of breath | | Fever |
|  | Somnolence during the day | | Memory problems | | Difficulties with daily activities |
|  | Motor Difficulties | | Coordination difficulties | | I do not present any symptom |
|  | Other:_____________________________________ | | | | |
| **Current Post-Covid Symptoms**  Check the currently persistent symptoms you had after the infection | Headache | | Olfactory changes | | Taste changes |
|  | Tiredness / Fatigue | | Shortness of breath | | Fever |
|  | Somnolence during the day | | Memory problems | | Difficulties with daily activities |
|  | Motor difficulties | | Coordination difficulties | | I do not present any symptom |
|  | Other:_____________________________________ | | | | |
| **Would you like to describe any more symptoms?** | (Your answer) | | | | |
| **May we contact you?** | Yes | | No | | |
| **If yes, do you have a preferred medium of contact?** | Registered e-mail | Registered phone | | Other | |

References

1 Brucki, S. M., Nitrini, R., Caramelli, P., Bertolucci, P. H. & Okamoto, I. H. [Suggestions for utilization of the mini-mental state examination in Brazil]. *Arq Neuropsiquiatr* **61**, 777-781, doi:10.1590/s0004-282x2003000500014 (2003).

2 Brucki, S. M. & Rocha, M. S. Category fluency test: effects of age, gender and education on total scores, clustering and switching in Brazilian Portuguese-speaking subjects. *Braz J Med Biol Res* **37**, 1771-1777, doi:10.1590/s0100-879x2004001200002 (2004).

3 Tombaugh, T. N., Kozak, J. & Rees, L. Normative data stratified by age and education for two measures of verbal fluency: FAS and animal naming. *Arch Clin Neuropsychol* **14**, 167-177 (1999).

4 Bolognani, S. A. P. *et al.* Development of alternative versions of the Logical Memory subtest of the WMS-R for use in Brazil. *Dement Neuropsychol* **9**, 136-148, doi:10.1590/1980-57642015dn92000008 (2015).

5 Weschsler, D. *Manual for the Wechsler Memory Scale - Revised.*, (TX: The Psychological Corporation, 1987).

6 Oliveira, M. S. & Rigoni, M. S. *Figuras complexas de Rey: teste de cópia e de reprodução de Memória de Figuras Geométricas Complexas*. 2ª ed edn, (Casa do Psicólogo, 2014).

7 Mathiowetz, V., Volland, G., Kashman, N. & Weber, K. Vol. 5 24-38 (The American journal of occupational therapy, 1985).

8 Sedo, M., Paula, J. J. & Malloy-Diniz, L. F. *Teste dos cinco dígitos*. (Hogrefe, 2015).

9 Rabelo, I. S. e. a. *Teste de trilhas coloridas*. (Casa do Psicólogo, 2018).

10 Gomes-Oliveira, M. H., Gorenstein, C., Lotufo Neto, F., Andrade, L. H. & Wang, Y. P. Validation of the Brazilian Portuguese version of the Beck Depression Inventory-II in a community sample. *Braz J Psychiatry* **34**, 389-394, doi:10.1016/j.rbp.2012.03.005 (2012).

11 de Lima Osório, F., Crippa, J. A. & Loureiro, S. R. Further psychometric study of the Beck Anxiety Inventory including factorial analysis and social anxiety disorder screening. *Int J Psychiatry Clin Pract* **15**, 255-262, doi:10.3109/13651501.2011.605955 (2011).

12 Chalder, T. *et al.* Development of a fatigue scale. *J Psychosom Res* **37**, 147-153, doi:10.1016/0022-3999(93)90081-p (1993).

13 Jackson, C. The Chalder Fatigue Scale (CFQ 11). *Occup Med (Lond)* **65**, 86, doi:10.1093/occmed/kqu168 (2015).

14 Bertolazi, A. N. *et al.* Portuguese-language version of the Epworth sleepiness scale: validation for use in Brazil. *J Bras Pneumol* **35**, 877-883, doi:10.1590/s1806-37132009000900009 (2009).

15 de Campos, B. M., Coan, A. C., Lin Yasuda, C., Casseb, R. F. & Cendes, F. Large-scale brain networks are distinctly affected in right and left mesial temporal lobe epilepsy. *Hum Brain Mapp* **37**, 3137-3152, doi:10.1002/hbm.23231 (2016).

16 Veraart, J., Fieremans, E. & Novikov, D. S. Diffusion MRI noise mapping using random matrix theory. *Magn Reson Med* **76**, 1582-1593, doi:10.1002/mrm.26059 (2016).

17 Veraart, J. *et al.* Denoising of diffusion MRI using random matrix theory. *Neuroimage* **142**, 394-406, doi:10.1016/j.neuroimage.2016.08.016 (2016).

18 Cordero-Grande, L., Christiaens, D., Hutter, J., Price, A. N. & Hajnal, J. V. Complex diffusion-weighted image estimation via matrix recovery under general noise models. *Neuroimage* **200**, 391-404, doi:10.1016/j.neuroimage.2019.06.039 (2019).

19 Tournier, J. D. *et al.* MRtrix3: A fast, flexible and open software framework for medical image processing and visualisation. *Neuroimage* **202**, 116137, doi:10.1016/j.neuroimage.2019.116137 (2019).

20 Kellner, E., Dhital, B., Kiselev, V. G. & Reisert, M. Gibbs-ringing artifact removal based on local subvoxel-shifts. *Magn Reson Med* **76**, 1574-1581, doi:10.1002/mrm.26054 (2016).

21 Schilling, K. G. *et al.* Synthesized b0 for diffusion distortion correction (Synb0-DisCo). *Magn Reson Imaging* **64**, 62-70, doi:10.1016/j.mri.2019.05.008 (2019).

22 Andersson, J. L. R. & Sotiropoulos, S. N. An integrated approach to correction for off-resonance effects and subject movement in diffusion MR imaging. *Neuroimage* **125**, 1063-1078, doi:10.1016/j.neuroimage.2015.10.019 (2016).

23 Andersson, J. L. R. *et al.* Towards a comprehensive framework for movement and distortion correction of diffusion MR images: Within volume movement. *Neuroimage* **152**, 450-466, doi:10.1016/j.neuroimage.2017.02.085 (2017).

24 Andersson, J. L. R., Graham, M. S., Drobnjak, I., Zhang, H. & Campbell, J. Susceptibility-induced distortion that varies due to motion: Correction in diffusion MR without acquiring additional data. *Neuroimage* **171**, 277-295, doi:10.1016/j.neuroimage.2017.12.040 (2018).

25 Smith, S. M. *et al.* Advances in functional and structural MR image analysis and implementation as FSL. *Neuroimage* **23 Suppl 1**, S208-219, doi:10.1016/j.neuroimage.2004.07.051 (2004).

26 Tustison, N. J. *et al.* N4ITK: improved N3 bias correction. *IEEE Trans Med Imaging* **29**, 1310-1320, doi:10.1109/TMI.2010.2046908 (2010).

27 Smith, S. M. *et al.* Tract-based spatial statistics: voxelwise analysis of multi-subject diffusion data. *Neuroimage* **31**, 1487-1505, doi:10.1016/j.neuroimage.2006.02.024 (2006).

28 Smith, S. M. & Nichols, T. E. Threshold-free cluster enhancement: addressing problems of smoothing, threshold dependence and localisation in cluster inference. *Neuroimage* **44**, 83-98, doi:10.1016/j.neuroimage.2008.03.061 (2009).

29 Winkler, A. M., Ridgway, G. R., Webster, M. A., Smith, S. M. & Nichols, T. E. Permutation inference for the general linear model. *Neuroimage* **92**, 381-397, doi:10.1016/j.neuroimage.2014.01.060 (2014).
